# Handling trial participants with missing outcome data when conducting a meta-analysis: a systematic survey of proposed approaches

**DOI:** 10.1186/s13643-015-0083-6

**Published:** 2015-07-23

**Authors:** Elie A. Akl, Lara A. Kahale, Thomas Agoritsas, Romina Brignardello-Petersen, Jason W. Busse, Alonso Carrasco-Labra, Shanil Ebrahim, Bradley C. Johnston, Ignacio Neumann, Ivan Sola, Xin Sun, Per Vandvik, Yuqing Zhang, Pablo Alonso-Coello, Gordon Guyatt

**Affiliations:** Department of Internal Medicine, Clinical Epidemiology Unit, American University of Beirut Medical Center, P.O. Box: 11–0236, Riad-El-Solh, Beirut, 1107, 2020 Beirut Lebanon; Department of Clinical Epidemiology and Biostatistics, McMaster University, Hamilton, Canada; Evidence-Based Dentistry Unit, Universidad de Chile, Santiago, Chile; Institute of Health Policy, Management and Evaluation, University of Toronto, Toronto, Canada; Department of Anesthesia and Pain Medicine, the Hospital for Sick Children, University of Toronto, Toronto, Ontario Canada; Department of Medicine, Stanford University, Stanford, CA USA; The Hospital for Sick Children Research Institute, Toronto, Ontario Canada; Institute of Health Policy, Management and Evaluation, Dalla Lana School of Public Health, University of Toronto, Toronto, Ontario Canada; Iberoamerican Cochrane Centre, Biomedical Research Institute Sant Pau (CIBERESP-IIB Sant Pau), Barcelona, Spain; The Norwegian Knowledge Centre for the Health Services, Oslo, Norway; Department of Medicine, McMaster University, Hamilton, Canada

**Keywords:** Missing participant data, Systematic reviews, Meta-analysis

## Abstract

**Background:**

When potentially associated with the likelihood of outcome, missing participant data represents a serious potential source of bias in randomized trials. Authors of systematic reviews frequently face this problem when conducting meta-analyses. The objective of this study is to conduct a systematic survey of the relevant literature to identify proposed approaches for how systematic review authors should handle missing participant data when conducting a meta-analysis.

**Methods:**

We searched MEDLINE and the Cochrane Methodology register from inception to August 2014. We included papers that devoted at least two paragraphs to discuss a relevant approach for missing data. Five pairs of reviewers, working independently and in duplicate, selected relevant papers. One reviewer abstracted data from included papers and a second reviewer verified them. We summarized the results narratively.

**Results:**

Of 9,138 identified citations, we included 11 eligible papers. Four proposed general approaches for handling dichotomous outcomes, and all recommended a complete case analysis as the primary analysis and additional sensitivity analyses using the following imputation methods: based on reasons for missingness (*n* = 3), relative to risk among followed up (*n* = 3), best-case scenario (*n* = 2), and worst-case scenario (*n* = 3). Three of these approaches suggested taking uncertainty into account. Two papers proposed general approaches for handling continuous outcomes, and both proposed a complete case analysis as the reference analysis and the following imputation methods as sensitivity analyses: based on reasons for missingness (*n* = 2), based on the mean observed in the same trial or other trials (*n* = 1), and based on informative missingness differences in means (*n* = 1). The remaining eligible papers did not propose general approaches but addressed specific statistical issues.

**Conclusions:**

All proposed approaches for handling missing participant data recommend conducting a complete case analysis for the primary analysis and some form of sensitivity analysis to evaluate robustness of results. Although these approaches require further testing, they may guide review authors in addressing missing participant data.

**Electronic supplementary material:**

The online version of this article (doi:10.1186/s13643-015-0083-6) contains supplementary material, which is available to authorized users.

## Background

Missing participant data (MPD) refers to participants excluded from the analysis of the primary study because no outcome data are available. MPD is a frequent problem in randomized clinical trials (RCTs) [1]. Karlson et al. found that the mean attrition rate reported in 40 trials of cognitive behavioral interventions in children with a chronic medical condition was 20 % for initial follow-up and 32 % for extended follow-up [13].

MPD may bias the effect estimates from RCTs when its occurrence is associated with the likelihood of outcome [1], and the risk of bias associated with MPD at the trial level is likely to translate into a similar risk at the meta-analysis level. Therefore, it is important that systematic review authors address MPD when conducting their meta-analyses and when assessing risk of bias.

The Cochrane handbook endorses two basic approaches to handling MPD: “available case analysis” and “analysis using imputations;” the Handbook authors classify the latter as an intention to treat analysis [17]. However, the handbook does not provide a detailed guidance on how to approach these analyses.

### Objective

The objective of this paper is to systematically survey the methodological literature to identify proposed approaches for how systematic review authors should handle MPD when conducting a meta-analysis.

## Methods

### Definition

From the perspective of a systematic review, missing participant data refers to the outcome data of trial participants that are not available to the reviewers (i.e., neither from the published trial reports nor from personal contact with trial authors) for inclusion in their meta-analyses. Missing data do not relate to missing studies (e.g., unpublished studies) or to unreported outcomes (e.g., outcomes planned in trial protocols but not included in trial reports).

### Eligibility criteria

We included English-language articles that devoted at least two paragraphs to discuss methods or conceptual approaches for how systematic reviews of RCTs could handle MPD for dichotomous and/or continuous outcomes. We excluded reports of systematic reviews and reports of original studies.

### Search strategy

We searched MEDLINE and the Cochrane Methodology register from their inception dates up to August 2014 using the OVID interface. An experienced researcher in developing literature search strategies (I.S.) developed the initial pilot search strategy. We refined the search strategy using relevant articles identified through the pilot search. Additional file 1 presents the detailed search strategy and Additional file 2 presents the PRISMA checklist.

### Article selection

Five pairs of reviewers trained in health research methodology conducted formal calibration exercises. These consisted of going through the same set of citations for the purpose of ensuring good understanding of eligibility criteria and the clarity of the instructions and forms before launching the formal screening process. Independently and in duplicate, the reviewers screened titles and abstracts, then, and full texts for eligibility using the web-based systematic review software (SRDistiller™). We used standardized piloted forms and detailed written instructions throughout the process to optimize agreement. Reviewers resolved disagreements by discussion and with the assistance of a third reviewer when needed.

### Data abstraction

One reviewer (L.K.) extracted data from included papers and a second reviewer (E.A.) verified the abstracted data. The remaining co-authors provided suggestions on how to improve data synthesis and presentation. We summarized our findings in both narrative and tabular formats.

### Data synthesis

We calculated agreement for the full text screening stage using the Kappa statistic. We judged the degree of agreement between pairs of reviewers and interpreted it according to Landis and Koch (*k* values of 0 to 0.20 represent slight agreement; 0.21 to 0.40, fair agreement; 0.41 to 0.60, moderate agreement; 0.61 to 0.80, substantial agreement; and greater than 0.80 values represent almost perfect agreement). We synthesized the data qualitatively and presented them in both narrative and tabular formats.

## Results

### Results of the search

Additional file 3 shows the study flow. Agreement between authors for study eligibility was almost perfect (kappa = 0.95). Out of 9138 citations, we identified 11 eligible papers reporting the following:

- Four general approaches for handling categorical missing data (*n* = 4 papers) [2, 10, 12, 14]
- Two general approaches for handling continuous missing data (*n* = 2) [9, 12]; (Note that Higgins 2008 addressed both categorical and continuous data).

The remaining papers addressed specific statistical issues for categorical missing data (*n* = 4 papers) [18–20, 22] and continuous missing data (*n* = 2 papers) [15, 16]. Among eight identified meeting abstracts, none addressed methods of handling continuous and categorical missing data of trial participants in systematic reviews.

### Findings

#### General approaches for categorical missing data

Table 1 summarizes the four proposed general approaches for handling MPD for dichotomous outcomes. Additional file 4 provides descriptions and illustration of analytical methods of dealing with missing participant data. All authors recommend a complete case analysis as a primary analysis, with additional sensitivity analyses using different imputation methods. Suggested imputation methods include the following: based on reasons for missingness [2, 10, 12], relative to risk among followed up participants [2, 12, 14], best-case scenario [10, 14], and worst-case scenario [2, 10, 14]. Three approaches suggest taking uncertainty into account [10, 12, 14]. Two papers suggested using their approaches to assess risk of bias associated with missing data [2, 14]. One paper tested its proposed approach through simulation [10], while the remaining three applied them to actual meta-analyses [2, 12, 14].

**Table 1:** Summary table of proposed general approaches for handling missing participant data for dichotomous outcomes

	Complete case analysis	Imputations for participants with missing outcome data					Take uncertainty into account	Relation with ITT principle	Assesses risk of bias associated with missing data	Testing of the proposed approach
		Based on reasons for missingness	Relative to risk among followed up	Best-case scenario	Worst-case scenario	Other imputation method				
Gamble and Hollis [10]	✓	✓	–	✓	✓	✓	✓	✓	–	✓
	As primary analysis (if missing data non-informative)	As primary analysis (if specified missing data mechanism)		As sensitivity analysis	As sensitivity analysis	Various separate imputations		Handling MPD needed for ITT analysis		Simulation study
Higgins et al. [12]	✓	✓	✓	–		–	✓	–	–	✓
	As primary analysis (point of reference)	As primary analysis (preferred)	(Using IMOR)							Applied in 1 meta-analysis of 17 RCTs
Akl et al. [2]	✓	✓	✓	–	✓	✓	–	✓	✓	✓
	As primary analysis	As primary analysis	Yes (using RI_LTFU/FU_)		As a way to assess risk of bias	Relative to observed incidence in trials included in meta-analysis		Handling MPD differentiated from ITT		Applied in 2 meta-analyses (with 20 and 22 RCTs, respectively)
Mavridis et al. [14]	✓		✓	✓	✓		✓		✓	✓
			(Using IMOR)^a^							Applied in one meta-analysis

RI_LTFU/FU_ refers to the event incidence among those lost to follow-up (LTFU) relative to the event incidence among those followed up (FU)

*ITT* intention to treat, *IMOR* informative missingness odds ratio

Of the four articles addressing specific statistical issues for categorical missing data [18–20, 22], one discussed correcting the bias resulting from missing data in a meta-analysis [22] and three related articles discussed statistical methods for allowing for uncertainty due to missing data in meta-analysis [18–20].

#### General approaches for continuous missing data

Table 2 summarizes two proposed general approaches for handling MPD for continuous outcomes [9, 12]. They both recommend a complete case analysis as a primary analysis and additional sensitivity analyses using different imputation methods, including based on reasons for missingness [9, 12], based on mean observed in the same trial [9], based on mean observed in the other trials [9], and based on informative missingness differences in means [12]. One approach suggests taking uncertainty into account [12].

**Table 2:** Summary table of proposed general approaches for handling missing participant data for continuous outcomes

	Complete case analysis	Imputations for participants with missing outcome data					Take uncertainty into account	Relation with ITT principle	Approach assesses risk of bias associated with missing data	Testing of the proposed approach
		Based on reasons for missingness	Based on mean observed in the same arm	Based on mean observed in the other arm	Based on mean observed in other included trials	Other imputation method				
Ebrahim et al. [9]	✓	✓	✓	✓	✓	–	–	–	✓	✓
	As primary analysis									Applied in 2 meta-analyses of 16 RCTs
Higgins et al. [12]	✓	✓		–		✓	✓	–	–	✓
	As primary analysis (point of reference)	As primary analysis (preferred)				Relative to risk among followed up; best and worst-case scenarios				Applied in 1 meta-analysis of 20 RCTs

Of the two articles addressing specific statistical issues for continuous missing data, one discussed pattern-mixed model which estimates summary effects while accounting for uncertainty in the outcome of the participants with missing outcome data [15] and one discussed the data according to the patterns of missing observations [16].

#### Description of individual approaches

Additional files 5 and 6 provide the recommendations of each included paper addressing categorical outcomes and continuous outcomes, respectively. The text in the additional files reproduces the paper’s own terminology for referring to MPD. Additional file 7 presents the definitions provided by each paper for the methods used to handle MPD in systematic reviews.

## Discussion

We have summarized the recommended approaches for how systematic review authors may handle MPD when conducting a meta-analysis. All general approaches recommend complete case analysis as the primary analysis. They also recommend additional sensitivity analyses using different imputation methods, mainly to assess the risk of bias associated with MPD. A commonly suggested approach is basing the imputation on the risk observed among followed up participants. Fewer approaches suggest taking uncertainty into account.

This is the first systematic survey addressing recommendations for the handling MPD in systematic reviews that we are aware of. Major strengths include explicit eligibility criteria, an exhaustive search, and systematic approaches to study selection, data abstraction, and data synthesis. One limitation of the review is the exclusion of non-English studies. Although focusing on English studies might lead to the loss of an appreciable number of eligible studies in clinical systematic reviews [7], this may be less of an issue for systematic surveys.

The different proposed approaches for dealing with missing participant data have advantages and disadvantages. The one proposed by Gamble and Hollis is the only one that has been tested using a simulation study. The approaches proposed by Higgins et al. and by Mavridis et al. relate the imputed odds of the outcome to its observed odds. The approach proposed by Akl et al. relates the imputed incidence of the outcome to its observed incidence and proposes a way to assess risk of bias associated with missing data.

The different analytical methods included in the above approaches have their own advantages and disadvantages.

- The complete case analysis method does not involve any imputations, making it the preferred choice in the main analysis. However, it typically results in loss in power, and it assumes that the missingness is due to reasons not related to the characteristics of these participants nor to the outcome of interest (missing completely at random assumption) [21].
- The best-case scenario and worst-case scenario methods represent implausible assumptions and cannot be used in the main analysis. However, the worst-case scenario might be useful in judging that the risk of bias associated with missing participant data as low, if its results (in a sensitivity analysis) do not substantially differ from those of the main analysis [2].
- Imputations using the informative missingness odds ratio (IMOR) and the RI_LTFU/FU_ have the advantage of basing the imputations on observed events. This makes their use reasonable when conducting sensitivity analyses to judge risk of bias associated with missing participant data. The main challenge is in determining the plausible values for these ratios.
- Any of the above imputations will increase the count of events and consequently narrow the confidence intervals of the effect estimate, implying increased certainty. However, this is misleading as the narrower confidence interval is based on imputed data. This makes the analytical method to handle uncertainty important to apply when using any of the above imputation methods.

We are not aware of rigorous studies evaluating or comparing different approaches and analytical methods of handling MPD in systematic review. While a large number of such studies have been published for trials [3, 11], their results do not directly inform the approach for systematic reviews. While trialists can use individual participant data to apply advanced statistical techniques such as multiple imputations [11], systematic reviewers can only use group level data with their inherent limitation, except in the case of individual participant data meta-analyses.

It is important to note the difference between a complete case analysis and per protocol analysis. Complete case analysis is intended to deal with the problem of participants with missing outcome data while per protocol analysis is intended to deal with the problem of non-compliant participants. The complete case analysis includes only participants with available outcome data. Per protocol analysis includes only participants who were compliant with the study protocol. The use of one analysis is independent of the use of the other. Indeed, Alshurafa et al. call for dealing with these two issues separately [4].

While the Cochrane Collaboration’s software (RevMan) does not include a module to account for missing data in meta-analysis, STATA has one for dichotomous data [8]. The “metamiss” command allows a complete case analysis as well as analyses applying a range of assumptions about the outcomes of participants with missing data [8]. It also applies the Gamble-Hollis analysis, which inflates the pooled effect estimate to reflect the uncertainty associated with missing data. Other software may have similar modules.

While the approaches we have identified require further testing, they may guide review authors facing missing participant data in their analysis. Systematic reviewers should also aim to minimize MPD by contacting the trialists to obtain unpublished but available data. In the unlikely case where trialists publish the outcomes of participants excluded from the trial analysis, the systematic reviewers may analyze them in the groups to which they were randomized.

The approaches presented in this systematic survey do require further empirical assessment. Indeed none of the imputation methods (including IMOR and RI) have been validated. Assessment could include simulation studies assessing the performance of the different approaches for handling MPD when conducting a meta-analysis, in relation to the truth [6]. Assessment could also compare the effect of the different approaches on pooled effect estimates, when applied to a sample of published systematic reviews. The findings of those investigations could then form the basis for consensus guidance on reporting, dealing with, and judging risk of bias associated with missing participant data in meta-analyses of randomized trials.

## Conclusions

Based on our findings, and pending further empirical evaluation, we suggest the following approach for handling MPD in a meta-analysis.

- First, calculate the best estimate of effect (primary analysis) using a complete case analysis.
- Then, assess the risk of bias associated with missing data by evaluating the robustness of the best estimate of effect (sensitivity analyses). These sensitivity analyses would consist of imputing the outcomes of participants with missing data using plausible assumptions.

The authors of systematic reviews can base the assumptions on reasons for missingness or estimate risks among participants with missing data relative to risk among those with available data. Further, approaches may (or may not) take uncertainty of the values attributed to the missing data into account.

## Additional files

Additional file 1:
**Search strategy.** Search strategy using Cochrane Methodology register and Ovid MEDLINE(R) In-Process and Other Non-Indexed Citations <1946 to Present >. Additional file 2:
**PRISMA 2009 Checklist.**
Additional file 3:
**PRISMA 2009 Flow Diagram.**
Additional file 4:
**Descriptions and illustration of analytical methods of dealing with missing participant data different sensitivity analyses of one trial.** Numerical data and results of different sensitivity analyses of one trial addressing perioperative anticoagulation in patients with cancer [5]. Additional file 5:
**Recommendations of each included paper addressing categorical outcomes.** The text here reproduces the paper’s own terminology for referring missing participant data terminology. Additional file 6:
**Recommendations of each included paper addressing continuous outcomes.** The text here reproduces the paper’s own terminology for referring missing participant data terminology. Additional file 7:
**Definitions provided for the methods used to handle MPD in systematic reviews.**

## Abbreviations

FU: follow-up
IMOR: informative missingness odds ratio
ITT: intention to treat
LTFU: lost to follow-up
MPD: missing participant data
RCTs: randomized clinical trials
RI: relative incidence
