# Supplementary material for: Handling trial participants with missing outcome data when conducting a meta-analysis: a systematic survey of proposed approaches
Source: Syst Rev. 2015 Jul 23;4:98. doi: 10.1186/s13643-015-0083-6 (PMC4511978; doi:10.1186/s13643-015-0083-6)
Supplement: Additional file 1: — Search strategy. Search strategy using Cochrane Methodology register and Ovid MEDLINE(R) In-Process and Other Non-Indexed Citations <1946 to Present >. [file 13643_2015_83_MOESM1_ESM.docx]

**Additional file 1:** Search Strategy

**MEDLINE**

#1 attrition.m_titl.

#2 "drop-out*".m_titl.

#3 missing.m_titl.

#4 "withdraw*".m_titl.

#5 (los* and follow*).m_titl.

#6 per protocol.m_titl.

#7 intention-to-treat.m_titl.

#8 intent-to-treat.m_titl.

#9 ITT.m_titl.

#10 (exclusion or exclusions).m_titl.

#11 excluded.m_titl.

#12 or/1-11

#13 ((randomized controlled trial or controlled clinical trial).pt. or randomized.ab. or placebo.ab. or drug therapy.fs. or randomly.ab. or trial.ab. or groups.ab.) not (exp animals/ not humans.sh.)

#14 12 and 13

**Cochrane Methodology Register/Cochrane Library**

attrition or (drop out) or missing or withdraw* or (loss* and follow*) or (per protocol) or (intention to treat) or (intent to treat) or ITT or (exclusion or exclusions or exclude):ti,ab,kw

Restricted to: Methods Studies and Technology Assessments
