# Supplementary material for: Handling trial participants with missing outcome data when conducting a meta-analysis: a systematic survey of proposed approaches
Source: Syst Rev. 2015 Jul 23;4:98. doi: 10.1186/s13643-015-0083-6 (PMC4511978; doi:10.1186/s13643-015-0083-6)
Supplement: Additional file 3: — PRISMA 2009 Flow Diagram. [file 13643_2015_83_MOESM3_ESM.docx]

**
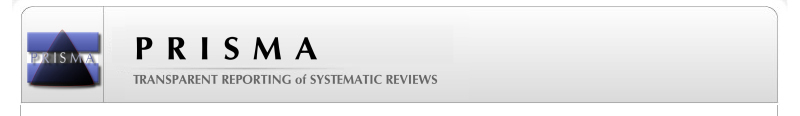
 Additional file** **3:** PRISMA 2009 Flow diagram

Records identified through database searching n= 10,572

- Medline n= 8622
- Cochrane methods register technology studies n= 19
- Cochrane methods register methods studies n= 1931

## Screening

## Eligibility

Additional records identified through other sources
n =0

Records after duplicates removed
n = 9138

Title and abstract screening
n =9138

Records excluded
n =8643

Full text screening
n = 495

N+

Full-text articles excluded, with reasons

n = 484

- meeting abstract n=8
- non-English n=1
- not about MPD n=139
- not about handling MPD in SR n=336

Eligible studies included
n =11

## Identification

## Included
