# Supplementary material for: Handling trial participants with missing outcome data when conducting a meta-analysis: a systematic survey of proposed approaches
Source: Syst Rev. 2015 Jul 23;4:98. doi: 10.1186/s13643-015-0083-6 (PMC4511978; doi:10.1186/s13643-015-0083-6)
Supplement: Additional file 4: — Descriptions and illustration of analytical methods of dealing with missing participant data different sensitivity analyses of one trial. Numerical data and results of different sensitivity analyses of one trial addressing perioperative anticoagulation in patients with cancer [5]. [file 13643_2015_83_MOESM4_ESM.docx]

**Additional file 4:** Descriptions and illustration of analytical methods of dealing with missing participant data different sensitivity analyses of one trial. Numerical data and results of different sensitivity analyses of one trial addressing perioperative anticoagulation in patients with cancer [5].

- *Complete case analysis:* includes only participants with available outcome data
- *Best case scenario:* assumes that all missing participants have a favourable outcome in the experimental group and poor outcome in the control group
- *Worst-case scenario:* assumes that all missing participants have a poor outcome in the experimental group and favourable outcome in the control group
- *IMOR / RI_LTFU/FU_ :*odds/ incidence among those lost to follow-up (LTFU) relative to the event odds/incidence among those followed up (FU)
- *Uncertainty:* Imputing the outcomes of participants with missing data will increase the total number of events and may result in narrowing the confidence intervals of the effect estimate. However, there is “uncertainty” associated with imputing outcomes that needs to be taken into account. This is done statistically for example by giving reduced weights to study with imputed outcomes to reflect the added uncertainty one might associate with data being missing. According to Gamble and Hollis, uncertainty interval can be calculated from the extreme confidence intervals of the best- and worst-case scenarios [11] .For example, if the confidence intervals of the best- and worst- case scenario of a certain trial are (0.3, 3.2) and (0.9, 19.1) respectively, then the uncertainty interval would be (0.3, 19.1). The authors suggested that this uncertainty interval incorporates the potential impact of the missing data as well as sampling error [11].

|  | Intervention group | | | Control group | | |
| --- | --- | --- | --- | --- | --- | --- |
| Trial | # Randomized | # Participant with MPD | # Observed events | # Randomized | # Participant with MPD | # Observed events |
| Bergqvist 1990 | 311 | 19 | 36 | 326 | 19 | 47 |

| Analytic method | Intervention group | | Control group | |
| --- | --- | --- | --- | --- |
|  | Numerator | Denominator | Numerator | Denominator |
| Complete case analysis | 36 | 311-19= 292 | 47 | 326-19= 307 |
| None of participants with MPD had the outcome of interest | 36 | 311 | 47 | 326 |
| All participants with MPD had the outcome of interest | 19 + 36= 55 | 311 | 19 + 47= 66 | 326 |
| Best case scenario | 36 | 311 | 19 + 47= 66 | 326 |
| Worst case scenario | 19 + 36= 55 | 311 | 47 | 326 |
| RI_LTFU/FU =_ 1:1 | [19 . ***1*** . 36 /(311 -19)] + 36= 39 | 311 | [19 . ***1*** . 47 /(326 -19)] + 47= 50 | 326 |
