# Supplementary material for: Handling trial participants with missing outcome data when conducting a meta-analysis: a systematic survey of proposed approaches
Source: Syst Rev. 2015 Jul 23;4:98. doi: 10.1186/s13643-015-0083-6 (PMC4511978; doi:10.1186/s13643-015-0083-6)
Supplement: Additional file 5: — Recommendations of each included paper addressing categorical outcomes. The text here reproduces the paper’s own terminology for referring missing participant data terminology. [file 13643_2015_83_MOESM5_ESM.docx]

**Additional File 5:** Recommendations of each included paper addressing categorical outcomes**.** The text here reproduces the paper’s own terminology for referring missing participant data terminology.

***Akl 2013[2]:***

This paper is a guide for systematic reviewers to deal with participants excluded from analyses of randomized trials who were considered ‘non-adherent to the protocol’ but for whom data are available, and participants with missing data. They first explain the relationship of MPD with Analysis-as-randomized (Intention to Treat Analysis). Then they suggest methods that aim to help in establishing the extent to which risk of bias impacts meta-analysis results. For excluded participants for whom data are available, they suggest ‘‘Intention to treat’’ analysis; ‘‘as treated’’ analysis; and ‘‘per protocol’’ analysis. For participants with missing data they recommend:

- Complete case analysis as a primary analysis
- Additional sensitivity analyses using different relatively extreme assumptions with variable degrees of plausibility to test the robustness of a result that is statistically significant, including:
  - Based on reasons for missingness,
  - Relative to risk among followed-up i.e. based on observed incidence in trials included in meta-analysis,
  - Worst-case scenario as a way to assess risk of bias,
  - Highest incidence among control arms of all included trials,
  - Highest incidence among intervention arms of all included trials.

They finally tested the proposed approach by applying it in two meta-analyses.

***Gamble 2005[11]:***

This paper suggests approaches to handling missing data in an intention-to-treat analysis in situations where little information on reasons for missingness is available. The authors recommend that a detailed breakdown of reasons for lack of outcome data should be provided. The following is suggested:

- Complete case analysis as primary analysis if missing data non informative,
- Uncertainty method with weights assigned to trials based on uncertainty interval widths alongside with complete case analysis,
- Best –worst case analysis,
- Based on reasons for missingness as primary analysis if specified missing data mechanism,
- Various separate imputations including simple, intermediate and multiple imputations.

The authors finally evaluated the performance of this method using simulated data.

***Mavridis 2014[15]:***

This paper presented methods used to account for missing outcome data in a systematic review and meta-analysis. First the authors discussed imputation of missing outcome data at the level of the individual trials, using two very common approaches replacing missing values with the mean value of the participants who provided data (simple imputation) and (2) replacing missing values with the last observed value. Then they described the challenges of several synthesis options to deal with missing outcome data at the level of the meta-analysis via an example of a systematic review comparing different antipsychotic medications for patients with schizophrenia:

- A complete case analysis leads to imprecise and potentially biased results.
- The best-case/worst case scenarios give unrealistic estimates,
- The uncertainty interval produces very conservative results,
- Imputation methods that replace missing data with values from the observed data do not properly account for the uncertainty introduced by the unobserved data and tend to underestimate standard errors,
- Employing a statistical model that links treatment effects in missing and observed data (Informative Missingness Odds Ratio - IMOR), unlike the other methods, reduces the weight assigned to studies with large missing rates.

Although this paper does not advocate certain methods, it presented an overview of most common approaches to deal with missing outcome data. It concluded that the differences between the results depend heavily on the rate of missing participants and that a sensitivity analysis is the only viable way to evaluate the effect of different scenarios for the missing data mechanism.

***White 2008[19, 20]:***

The authors discussed in the 2 papers (part 1 and part 2) statistical methods for allowing for uncertainty due to missing data in meta-analysis. In order to appropriately capture the extra uncertainty due to missing data, they suggest a realistic prior for IMORs, including an assessment of the prior correlation between IMORs in different arms and in different trials. The authors considered that clinical members are the best available source of prior beliefs since they have the required subject knowledge without having personal involvement in the trials that could lead them to be over-optimistic. Four sensitivity analyses are proposed:

- IMORs assumed fixed for all trials and equal across arms,
- IMORs assumed fixed for all trials and opposite across arms,
- IMORs assumed random, independent across trials and equal across arms,
- IMORs assumed random, independent across trials and uncorrelated across arms,
- They illustrate the strategy proposed for sensitivity analysis using one meta-analysis.

***Yuan 2008[22]:***

This paper describes a statistical method for the specific case where primary study has made certain missing data adjustments so that the reported estimates of treatment effect size and variance are valid. The authors propose three methods to correct the bias resulting from meta-analysis based on the standard random-effects model:

- Reweighting the DerSimonian–Laird estimate by the completion rate,
- Incorporating the completion rate into a Bayesian random-effects model (Reweighted Bayesian random-effects (RWRE) model),
- Inference based on a Bayesian shared-parameter model that jointly model the outcome and missing data mechanism,

***Higgins 2008[13]:***

The authors propose a strategy for addressing missing binary outcome data from trials included in a meta-analysis:

- Based on reasons for missingness as preferred primary analysis,
- Complete case analysis as primary analysis (point of reference),
- Relative to risk among followed-up using IMOR,
- Taking uncertainty into account.

The principles behind the proposed strategy are precision, reduction of bias, scale independence, and simplicity. The authors tested the approach in one meta-analysis of 20 RCTs.

***Turner 2015[18]:***

This paper proposes a conceptually simple Bayesian framework to account for uncertainty associated with missing binary outcome data in meta-analysis. A pattern-mixture model is fitted, which allows the incorporation of prior information on a parameter describing the missingness mechanism. The paper explores the ability of the model to learn about the missingness parameters using artificial data scenarios. The authors used as an example a meta-analysis of 17 RCTs comparing haloperidol versus placebo for schizophrenia with missing binary outcome data.
