# Supplementary material for: Handling trial participants with missing outcome data when conducting a meta-analysis: a systematic survey of proposed approaches
Source: Syst Rev. 2015 Jul 23;4:98. doi: 10.1186/s13643-015-0083-6 (PMC4511978; doi:10.1186/s13643-015-0083-6)
Supplement: Additional file 6: — Recommendations of each included paper addressing continuous outcomes. The text here reproduces the paper’s own terminology for referring missing participant data terminology. [file 13643_2015_83_MOESM6_ESM.docx]

**Additional File 6:** Recommendations of each included paper addressing continuous outcomes. The text here reproduces the paper’s own terminology for referring missing participant data terminology

***Ebrahim 2013[10]:***

This paper aims to develop a framework for handling missing participant data for continuous outcomes. The authors considered sources reflecting real observed outcomes in participants followed-up in individual trials included in the systematic review:

- The best mean score among the intervention arms of the included trials,
- The best mean score among the control arms of the included trials,
- The mean score from the control arm of the same trial,
- The worst mean score among the intervention arms of the included trials,
- The worst mean score among the control arms of the included trials.

The authors suggest an approach that involves an initial complete case analysis with subsequent sensitivity analyses making progressively more stringent assumptions about results in patients with missing data:

- Strategy 1 uses the mean score from the control arm of the same trial for those with missing data in both the intervention and control arm,
- Strategy 2 uses the worst mean score among the intervention arms of the included trials for those with missing data in the intervention arm, and the best mean score among the control arms of the included trials for those with missing data in the control arm,
- Strategy 3 uses the worst mean score among the control arms of the included trials for those with missing data in the intervention arm, and the best mean score among the control arms of the included trials for those with missing data in the control arm,
- Strategy 4 uses the worst mean score among the control arms of the included trials for those with missing data in the intervention arm, and the best mean score among the intervention arms of the included trials for those with missing data in the control arm.

The approach was applied to two systematic reviews.

***Talwalker 1996 [17]:***

This paper describes a specific statistical method of stratifying the data according to the patterns of missing observations, summarizing each subject’s repeated measurements by a summary measure and then comparing the treatment groups with the help of a distribution-free test based on the summary measure.

***Higgins 2008 [13]:***

The authors propose a strategy for addressing missing continuous outcome data from trials included in a meta-analysis:

- Based on reasons for missingness as preferred primary analysis,
- Complete case analysis as primary analysis (point of reference),
- Relative to risk among followed-up using ‘informative missingness difference in means or informative missingness ratio of means to impute missing outcomes that are similar, bigger or smaller than the observed outcomes within any particular treatment group,
- Taking uncertainty into account.

The principles behind the proposed strategy are precision, reduction of bias, scale independence, and simplicity. The authors tested the approach in one meta-analysis of 20 RCTs.

***Mavridis 2014 [16]***

This paper declares that estimated treatment effects based on complete case analysis are potentially biased if informative missing data are ignored. Thus the authors propose a new model “pattern-mixture model” which quantifies the degree of departure from missing at random using either an informative missingness difference of means (IMDoM) or an informative missingness ratio of means (IMRoM) , both of which relate the mean value of the missing outcome data to that of the observed data.

To account for uncertainty in the outcome of participants with missing data, the model proposes estimating the treatment effects, adjusted for informative missingness, and their standard errors by a Taylor series approximation and by a Monte Carlo method. The methodology is applied on examples of both pairwise and network meta-analysis with multi-arm trials. The paper concludes that as missingness parameters increases, CIs for the study estimates increasingly overlap and estimated heterogeneity consequently reduces, leading to increased uncertainty in the summary result.
