# Supplementary material for: Handling trial participants with missing outcome data when conducting a meta-analysis: a systematic survey of proposed approaches
Source: Syst Rev. 2015 Jul 23;4:98. doi: 10.1186/s13643-015-0083-6 (PMC4511978; doi:10.1186/s13643-015-0083-6)
Supplement: Additional file 7: — Definitions provided for the methods used to handle MPD in systematic reviews. [file 13643_2015_83_MOESM7_ESM.docx]

**Additional File 7:** Definitions provided for the methods used to handle MPD in systematic reviews.

***Akl 2013[2]:***

*Missing participant data:* data unavailable to the investigator(s) or available to the investigator(s) but not included in published reports

*Intention to treat analysis:* when calculating a relative risk for a trial, the total of the excluded participants is added to the denominator and the number with events is added to the numerator of the arm to which they were randomized

*As treated analysis*: the total excluded participants and the number with events are added, respectively, to the denominator and numerator of the arm of the intervention that they actually received*.*

*Per protocol analysis:* ‘non-adherent’ participants remain excluded from the analysis (i.e., their total number and the number with events are not included in the denominator or numerator respectively)

*Complete case analysis* or *available case analysis*: participants with missing data are excluded from both the numerator and denominator when calculating relative risk of a trial

*RI_LTFU/FU_*_:_ refers to the relative incidence among those with missing data (lost to follow-up; LTFU) compared to those with available data in the same arm (followed-up; FU)

***Gamble and Hollis 2005[11]:***

*Available case analysis* (complete case analysis): includes only those participants whose outcome data are known

*Imputed case analysis*: missing values are filled in using specific assumptions about what might have happened to the participants

*Best–worst case analysis:* where the two most extreme imputations are considered

*Informative missingness odds ratio (IMOR*): risk among the missing participants in the form of the odds ratio of the event among missing participants relative to the event among observed participants / the relative risks among missing and observed participants

*Uncertainty*: a simplistic ad hoc approach that decreases the amount of weight given to studies with missing data. The weighting is based on the amount of information contained in each trial, taking into consideration both the sampling error and the potential impact of the missing data

***Mavridis 2014[15]:***

*Complete cases meta-analysis:* only individuals whose outcome is known are included.

*Best-case and worst-case scenario:* The best-case scenario assumes that all missing participants have a favourable outcome in the experimental group and poor outcome in the control group; the converse is assumed for the worst-case scenario.

*Uncertainty intervals:* Studies for which there is a big discrepancy between best-case and worst-case scenarios should be down-weighed. Best-worst case scenarios give an interval, called uncertainty interval, for the treatment effect that includes all uncertainty due to missing data. A pseudo SE is estimated from this for each study and is subsequently used to down-weigh studies. A summary estimate is computed using the revised weights from the uncertainty intervals. This method has low power when there is a large amount of missing data.

*IMOR:* describes the relationship between the unknown odds of the outcome among missing participants and the known odds among observed participants.

***White 2008[19,20]:***

*Informative missingness (IM):* the degree of departure from the “missing at random” assumption

*Bayesian pattern-mixture model:* comprises the probability of the outcome being missing, and a model for the outcome conditional on whether or not it is missing. IM is quantified as a contrast of the (unobserved) success probability in the missing data and the success probability in the observed data. Thus, the parameters that are estimable from the data (the missing data fraction and the success fraction in the observed data) are explicitly distinguished from those that are not estimable from the data (the success fraction in the missing data). Uncertainty of missing data is incorporated using a suitable prior distribution for the degree of IM.

*Worst-case approach*: assumes that all missing data are failures in the intervention trial arm and successes in the control arm,

*Best-case approach:* assumes that all missing data are failures in the control trial arm and successes in the intervention arm,

***Yuan 2008[22]:***

*Missing data at the individual patient level:* such as nonresponse or failure to record patient outcomes

*Fixed-effects model:* assumes that all the studies estimate a common underlying treatment effect

*Random-effect model:* accounts for heterogeneity among studies by assuming that treatment effect is not fixed, but is itself random and has its own distribution.

***Ebrahim 2013[10]:***

*Missing participant data:* data unavailable to the investigator(s) or available to the investigator(s) but not included in published reports

***Talwalker 1996[17]:***

*Intention to treat population:* consisted of all patients who were randomized, took at least one dose of the drug, and had at least one post treatment measurement

*Distribution-free test:* stratification of data according to the patterns of missing observations, calculation of a nonparametric test statistic based on the summary measure for the comparisons of two groups in each stratrum, and testing the hypothesis of overall equality of the distribution of summary statistic in two groups using a weighted linear combination of the stratum -specific test statistics

***Mavridis 2014[16]:***

*Missing at random (MAR):* the probability of data being missing does not depend on the outcome within randomized groups

*Informatively Missing Odds Ratio (IMOR):* the ratio of the odds of success in the missing data to the corresponding odds in the observed data

*Pattern-mixture model:* makes hypotheses for the unobserved data given the observed data, i.e., the degree of departure from the MAR assumption is quantified by the informative missingness odds ratio (IMOR). It enables estimation of summary effects while accounting for uncertainty in the outcome of the participants who dropped out.

***Higgins 2008[13]:***

*Missing completely at random*: missingness of an outcome is not related to any observed or unobserved variables

*Missing at random*: missingness of an outcome may be related to observed or unobserved variables, but is not related to the actual value of the outcome, conditional on the observed variables

*Informatively missing*: missingness of an outcome is related to the value of that outcome, even conditional on other observed variables
